# Balanced truncation for model reduction of biological oscillators

**DOI:** 10.1007/s00422-021-00888-4

**Published:** 2021-08-12

**Authors:** Alberto Padoan, Fulvio Forni, Rodolphe Sepulchre

**Affiliations:** Department of Engineering, University of Cambridge, Cambridge, UK

**Keywords:** Model reduction, Balanced truncation, Dominance theory, Biological oscillators

## Abstract

Model reduction is a central problem in mathematical biology. Reduced order models enable modeling of a biological system at different levels of complexity and the quantitative analysis of its properties, like sensitivity to parameter variations and resilience to exogenous perturbations. However, available model reduction methods often fail to capture a diverse range of nonlinear behaviors observed in biology, such as multistability and limit cycle oscillations. The paper addresses this need using differential analysis. This approach leads to a nonlinear enhancement of classical balanced truncation for biological systems whose behavior is not restricted to the stability of a single equilibrium. Numerical results suggest that the proposed framework may be relevant to the approximation of classical models of biological systems.

## Introduction

Model reduction is a central problem in mathematical biology  (Fall et al. 2005; Murray 2007; Alon 2007; Keener and Sneyd 2008; Del Vecchio and Murray 2015). Besides their role in numerical simulations, models enable the study of the principles underlying the behavior of a biological system and the quantitative analysis of its properties, like sensitivity to parameter variations and resilience to exogenous perturbations. Models of biological phenomena often originate from complex networks of chemical reactions (Fall et al. 2005; Murray 2007; Alon 2007; Keener and Sneyd 2008; Del Vecchio and Murray 2015), the temporal dynamics of which is modeled by possibly large systems of differential equations. The resulting nonlinear dynamical models pose significant challenges for simulation, analysis and design. Model reduction alleviates these issues by constructing reduced order models whose behavior captures that of the original system (Antoulas 2005). Motivated by the goal of designing biological devices of increasing size and complexity (Del Vecchio et al. 2018), model reduction of biological systems has recently attracted renewed interest in the rapidly emerging field of systems and synthetic biology (Gómez-Uribe et al. 2008; Anderson et al. 2011; Thomas et al. 2012; Kang and Kurtz 2013; Rao et al. 2014; Sootla and Anderson 2014; Radulescu et al. 2015; Del Vecchio and Murray 2015; Herath et al. 2016; Sootla and Anderson 2017; Herath and Del Vecchio 2018). Given the pressing need for compositional modeling frameworks in mathematical biology (Del Vecchio et al. 2018), the present paper seeks to develop a model reduction framework compatible with modeling and interconnection of open systems whose behavior is not restricted to the stability of a single equilibrium.

*Background* Model reduction of biological systems has a long history (Snowden et al. 2017), with early contributions dating back as far as the 1920s (Briggs and Haldane 1925). The classical approach to model reduction of biological systems relies on *timescale separation* arguments (Briggs and Haldane 1925; Segel and Slemrod 1989; Gómez-Uribe et al. 2008; Thomas et al. 2012; Kang and Kurtz 2013; Prescott and Papachristodoulou 2014; Del Vecchio and Murray 2015; Herath et al. 2016; Herath and Del Vecchio 2018). In general, many biochemical processes are described by reactions that evolve on different timescale, which enables the separation of their dynamics into “slow” and “fast.” This property is exploited by methods based on timescale separation to reduce the complexity of a system by approximating the fast variables with their steady-state values. This approach preserves the biological meaning of the state space variables and favors modularity between processes with widely separated timescales (Grunberg and Del Vecchio 2019). However, timescale separation methods are only applicable when the underlying biochemical reaction network behaves like a *closed system* and may otherwise yield reduced order models whose behavior is qualitatively different from that of the original system (Stoleriu et al. 2004, 2005; Flach and Schnell 2006; Pedersen et al. 2008). A popular alternative to methods based on timescale separation is *lumping* (Wei et al. 1969; Tomlin et al. 1994; Koschorreck et al. 2007; Sunnåker et al. 2011; Rao et al. 2014), which aggregates state space variables into “macroscopic” variables to reduce the dimensionality of a system. While in some cases this maintains biological interpretability and compatibility with open systems modeling (Rao et al. 2014), lumping often requires expert knowledge, which makes it unappealing for constructive design and quantitative verification. Another common approach to model reduction of biochemical systems is based on *sensitivity analysis* and *optimization* (Danø et al. 2006; Zhang and Goutsias 2010; Prescott and Papachristodoulou 2012; Hangos 2013), which build reduced order models by minimizing an error function (such as the sensitivity of a variable) within a given range of candidate models. Despite its wide applicability, this approach can be highly demanding from a computational viewpoint for large-scale models and, in general, offers no *a priori* guarantees on the behavior of the reduced order model. A notable exception is given by methods based on *balanced truncation* (Liebermeister et al. 2005; Hardin and van Schuppen 2006; MeyerBase and Theis 2008; Sootla and Anderson 2014) and on the use of related *Linear Matrix Inequalities* (LMIs) (Anderson et al. 2011). In broad terms, balanced truncation first computes a change of coordinates in which the degrees of reachability and observability of each state are the same; the states which are least controllable and observable are then truncated to obtain a reduced order model. A nice feature of balanced truncation is that it preserves stability and that it offers *a priori* error bounds in the $\mathcal {L}_2$ norm. However, balanced truncation is primarily a linear method and, hence, is not directly applicable to capturing important nonlinear behaviors observed in biology, such as multistability and limit cycle oscillations (Fall et al. 2005; Murray 2007; Alon 2007; Keener and Sneyd 2008; Del Vecchio and Murray 2015). Motivated and inspired by this line of research, the present paper extends the applicability of balanced truncation to behaviors that are not restricted to the stability of a single equilibrium.

The model reduction problem has been extensively studied also in the systems and control literature (Antoulas 2005). The problem is well understood for finite-dimensional, linear, time-invariant systems, for which standard methods are based on *balanced truncation* (Moore 1981; Glover 1984), on *moment matching* (Georgiou 1983; Kimura 1986; Antoulas et al. 1990; Georgiou 1999), and on a combination of both approaches (Gugercin and Antoulas 2006; Gugercin 2008; Padoan). Oscillatory behaviors can be approximated, in principle, using balanced truncation for linear periodic systems (Varga 2000) and linear time-varying systems (Sandberg and Rantzer 2004). However, both approaches lead to reduced models whose state space dimension may vary over time and which are thus not amenable to analysis and design. Several methods exist to approximate the *local* behavior of nonlinear, time-invariant systems around equilibrium (Antoulas 2005), including *proper orthogonal decomposition* (Berkooz et al. 1993), *balanced truncation* (for stable input-affine systems) (Scherpen 1993), *empirical balanced truncation* (Hahn and Edgar 2002), *moment matching* (Astolfi 2010; Padoan and Astolfi 2019) *discrete empirical interpolation* (Chaturantabut and Sorensen 2010), *high-order moment matching* (Asif et al. 2020) and $\mathcal {H}_2$-*optimal model reduction* (Benner et al. 2018). However, these methods do not provide *a priori* guarantees on the *global* behavior of reduced order models away from equilibrium, which limits their applicability to multistable and oscillatory systems. The problem of approximating the global behavior of a nonlinear system is indeed largely open. Besselink and co-authors have recently proposed a model reduction framework which preserves (incremental) stability properties for systems that can be decomposed as the feedback interconnection of a large-scale stable linear system and a *contractive* nonlinear system (Besselink et al. 2009, 2013, 2014). In the recent papers (Padoan et al. 2021, 2020), we have generalized these results to multistable and oscillatory systems that can be decomposed as the feedback interconnection of a large-scale stable linear system and a *dominant* nonlinear system. The present paper extends our preliminary results to general dominant systems motivated by the increasing need of approximation tools for biological systems away from equilibrium.

*Contributions* This paper proposes a model reduction framework for biological systems whose behavior is not restricted to the stability of a single equilibrium. Motivated and inspired by the series of works (Besselink et al. 2009, 2013, 2014) and our preliminary results (Padoan et al. 2020, 2021), the present paper revisits the model reduction problem for nonlinear systems in light of *differential analysis* and *dominance theory* (Forni and Sepulchre 2019; Miranda-Villatoro et al. 2018; Padoan et al. 2019a, b, 2020, 2021). A nonlinear enhancement of classical balanced truncation for linear stable systems is developed for *p*-*dominant* systems. Following the paradigm of classical balanced truncation, the model reduction problem is reduced to the simultaneous diagonalization of a pair of gramian matrices, computed using the linearization of a system and LMIs subject to a fixed inertia constraint. The asymptotic behavior of a reduced order model is then characterized by ensuring that the property of *p-dominance* is preserved.

Although the biological meaning of state space variables is not necessarily preserved, our approach offers a series of benefits. First, it favors *tractability* leveraging on standard convex optimization tools to build reduced order models. Second, the quality of a reduced order model can be interpreted in *quantitative* terms using classical control-theoretic notions and tools, such as eigenvalues, Nyquist diagrams and Bode diagrams. Third, it provides a *compositional* model reduction framework, where the global emergent behavior of a complex biological system can be approximated using reduced order models of its elementary components. As a motivating example, we consider the problem of approximating the Goldbeter model for the expression of the *per* gene in *Drosophila* (Goldbeter 1995)—a paradigmatic example of biological oscillations (see Goldbeter 1996; Fall et al. 2005; Murray 2007; Keener and Sneyd 2008). Numerical results suggest that the proposed framework may be relevant to the approximation of a wide range of biological systems.

*Paper organization* The remainder of this work is organized as follows. Section 2 first recalls preliminary results on balanced truncation for stable linear systems and on dominance theory. Classical balanced truncation is then revisited in light of dominance theory to develop a model reduction method for the analysis of multistable and oscillatory systems. Section 3 illustrates the applicability of the proposed model reduction framework by means of a worked-out numerical example. Section 4 discusses main benefits and possible improvements of the proposed method. Section 5 concludes the paper with a summary of our main results and an outlook to future research directions. The Appendix contains more detail on the algorithms behind parameter selection for dominance analysis and simultaneous diagonalization of matrices with a fixed inertia.

*Notation* $\mathbb {R}$ and $\mathbb {C}$ denote the set of real numbers and the set of complex numbers, respectively. $\mathbb {R}_{+}$ denotes the set of nonnegative real numbers. ${\sigma ({A})}$ denotes the spectrum of the matrix ${A \in \mathbb {R}^{n \times n}}$. The inertia of the matrix ${P \in \mathbb {R}^{n \times n}}$ is defined as ${\text {In}}(P) = (n_{-},n_{0},n_{+})$, where $n_{-}$ is the number of eigenvalues of *P* in the open left half plane, $n_{0}$ is the number of eigenvalues of *P* on the imaginary axis and $n_{+}$ is the number of eigenvalues of *P* in the open right half plane, respectively.

## Methods

### Balanced truncation for linear stable systems

Consider a linear, time-invariant, system described by the equations

1 $$\begin{aligned} \quad \dot{x} = Ax+Bu, \quad y=Cx, \end{aligned}$$

in which ${x \in \mathbb {R}^n}$, ${u\in \mathbb {R}^m}$, ${y\in \mathbb {R}^l}$, ${A\in \mathbb {R}^{n \times n}}$, ${B\in \mathbb {R}^{n \times m}}$, ${C\in \mathbb {R}^{l\times n}}$, respectively. Assume that system (1) is stable and minimal, i.e, reachable and observable.

#### Balancing

*Balancing* for system (1) consists in finding a coordinates transformation ${\bar{x} = T^{-1} x }$, with ${T\in \mathbb {R}^{n \times n}}$ and ${\det (T) \not = 0}$, such that the *reachability gramian* ${P\in \mathbb {R}^{n \times n}}$ and the *observability gramian* ${Q\in \mathbb {R}^{n \times n}}$, defined implicitly by the Lyapunov equations

2 $$\begin{aligned} A P +PA^{\mathsf {T}} +BB^{\mathsf {T}}&= 0, \end{aligned}$$

3 $$\begin{aligned} A^{\mathsf {T}} Q +QA +C^{\mathsf {T}}C&= 0, \end{aligned}$$

are both diagonal and, if possible, equal. Stability of system (1) ensures existence and positive definiteness of the gramians, while minimality ensures that these are full rank (Antoulas 2005).

A change of coordinates ${\bar{x} = T^{-1} x}$ for system (1) acts on the reachability gramian and the observability gramian as

4 $$\begin{aligned} \bar{P} = T^{-1} P T^{-\mathsf {T}}, \quad \bar{Q} = T^{\mathsf {T}} Q T. \ \end{aligned}$$

Balancing thus amounts to finding a transformation *T* which *simultaneously diagonalizes* the positive definite matrices *P* and *Q*. This is a classical problem in linear algebra with a well-known solution (Bernstein 2009, p. 422).

The matrix $\{T\in \mathbb {R}^{n \times n}\}$ is said to be a *(principal-axis) balancing transformation* if

5 $$\begin{aligned} \bar{P} = \bar{Q} = \Sigma = {\text {diag}}(\sigma _1, \ldots , \sigma _n), \end{aligned}$$

in which case the corresponding representation in coordinates of system (1) is said to be *(principal-axis) balanced* (Antoulas 2005). The diagonal elements of $\Sigma$ are referred to as the *Hankel singular values* of the system. The Hankel singular values do not depend on the particular coordinate system and their magnitude quantifies the influence of each state on the overall input–output behavior of the system (Antoulas 2005).

#### Model reduction by balanced truncation

*Balanced truncation* for system (1) consists in eliminating, by truncation, the state variables corresponding to the least $n-r$ Hankel singular values, where ${r \le n}$ is the order of the reduced order model. The resulting reduced order model

6 $$\begin{aligned} \quad \dot{\xi } = {\hat{A}}\xi +{\hat{B}}{\hat{u}}, \quad {\hat{y}}={\hat{C}}\xi , \end{aligned}$$

with ${\xi \in \mathbb {R}^r}$, ${{\hat{u}}\in \mathbb {R}^m}$, ${{\hat{y}}\in \mathbb {R}^l}$, is guaranteed to be stable and to satisfy the $\mathcal {L}_{2}$ error bound^1^

7 $$\begin{aligned} \left\Vert y - {\hat{y}}\right\Vert _{2} \le \left( \textstyle 2 \sum _{j={ r}+1}^{n} \sigma _{j} \right) \left\Vert u\right\Vert _{2} \end{aligned}$$

for all $${u \in \mathcal {L}_{2}}$$.

While classical balanced truncation only applies to linear, time-invariant, systems, several nonlinear extensions exist (see, e.g., Scherpen 1993; Hahn and Edgar 2002; Besselink et al. 2009, 2013, 2014; Benner et al. 2018). All those references however consider stable nonlinear systems with a unique equilibrium attractor, except for (Benner et al. 2018), which, however, does not provide *a priori* guarantees on the *global* behavior of reduced order models away from equilibrium. The goal of the present paper is to propose model reduction methods for nonlinear systems with multiple stable equilibria or stable limit cycle attractors. The proposed approach is based on recent results from dominance theory, which we discuss in the next section.

### Dominance theory

Consider a nonlinear, time-invariant system and its variational dynamics described by the equations

8 $$\begin{aligned} \quad \ \dot{x}&= f(x) +Bu, \quad \quad \quad \ \ \ y = \ Cx , \end{aligned}$$

9 $$\begin{aligned} \quad \dot{\delta x}&= \partial f(x) \delta x + B \delta u, \quad ~\, \delta y =~ C \delta x , \end{aligned}$$

in which ${x\in \mathbb {R}^n}$, ${u\in \mathbb {R}^m}$, ${y\in \mathbb {R}^l}$, ${f:\mathbb {R}^{n} \rightarrow \mathbb {R}^{n}}$ is a continuously differentiable vector field, ${B\in \mathbb {R}^{n\times m}}$ and $C\in \mathbb {R}^{l\times n}$ are constant matrices, ${\delta x\in \mathbb {R}^n}$, ${\delta u\in \mathbb {R}^m}$, ${\delta y\in \mathbb {R}^l}$ (identified with the respective tangent spaces), and ${\partial f}$ is the Jacobian of the vector field *f*.

#### Definition 1

(Forni and Sepulchre 2019) The system (8) is said to be *p*-dominant with rate ${\lambda : \mathbb {R}^n \rightarrow \mathbb {R}_{+}}$ if there exist ${\varepsilon \in \mathbb {R}_{+}}$ and a symmetric matrix ${P\in \mathbb {R}^{n\times n}}$, with inertia ${\text {In}}(P) = (p, 0,n-p)$, such that the prolonged system (8)-(9) satisfies

10 $$\begin{aligned} \left[ \begin{array}{c} \dot{\delta x} \\ \delta x \end{array} \right] ^{\mathsf {T}} \left[ \begin{array}{cc} ~0~\, &{} ~P \\ ~P~\, &{} ~2\lambda (x) P + \varepsilon I~ \end{array} \right] \left[ \begin{array}{c} \dot{\delta x} \\ \delta x \end{array} \right] \le 0 \end{aligned}$$

for all ${(x,\delta x) \in \mathbb {R}^n \times \mathbb {R}^n}$ and for ${\delta u =0}$. The property is strict^2^ if ${\varepsilon >0}$.

The property of *p*-dominance yields the quadratic form

11 $$\begin{aligned} V(\delta x) = \delta x^{\mathsf {T}} P \delta x \end{aligned}$$

which allows one to rewrite the inequality (10) as

12 $$\begin{aligned} \dot{V}(\delta x) \le - 2\lambda (x) V(\delta x) - \varepsilon |\delta x|^2. \end{aligned}$$

The quadratic form $V(\delta x)$ thus characterizes the contraction of the cones $\mathcal {K}_+ = \{\, \delta x \in \mathbb {R}^{n-p} \,:\, V(\delta x) \ge 0 \,\}$ and $\mathcal {K}_- = \{\, \delta x \in \mathbb {R}^{p} \,:\, V(\delta x) \le 0 \,\}$ acting as a Lyapunov function for the variational dynamics of the system in forward and backward time, respectively. The solutions of the linearization of a *p*-dominant system thus split into ${n-p}$ *transient* modes and *p* *dominant* modes, which determine the asymptotic behavior of the system. The attractors of a *p*-dominant system are therefore severely constrained for small values of *p* (Forni and Sepulchre 2019).

#### Theorem 1

(Attractors of dominant systems) Assume system (8) is *p*-dominant with rate ${\lambda : \mathbb {R}^n \rightarrow \mathbb {R}_{+}}$. Then, for any constant input *u*, every bounded solution of (8) converges asymptotically to

- the unique equilibrium point if ${p=0}$;
- a (possibly non-unique) equilibrium point if ${p=1}$;
- a simple attractor if ${p=2}$, *i.e.*, an equilibrium point, a set of equilibrium points and their connected arcs or a limit cycle.

For further detail on dominance theory, the reader is referred to the paper (Forni and Sepulchre 2019).

### Differential balanced truncation for dominant nonlinear systems

#### Differential balancing

*Differential balancing* for system (8) consists in finding a change of coordinates ${\bar{x} = T^{-1} x}$, with $T\in \mathbb {R}^{n \times n}$ such that $\det T \not = 0$, such that the reachability gramian ${P\in \mathbb {R}^{n \times n}}$ and the observability gramian ${Q\in \mathbb {R}^{n \times n}}$, defined implicitly by the Lyapunov inequalities

13 $$\begin{aligned}&\partial f(x) P +P\partial f(x)^{\mathsf {T}} + 2\lambda (x) P +BB^{\mathsf {T}} +\varepsilon I \le 0, \end{aligned}$$

14 $$\begin{aligned}&\partial f(x)^{\mathsf {T}} Q +Q\partial f(x) + 2\lambda (x) Q +C^{\mathsf {T}}C +\varepsilon I \le 0, \end{aligned}$$

for ${x\in \mathcal {S} \subset \mathbb {R}^n}$, are both diagonal and, if possible, equal.

Differential balancing is conceptually similar to classical balancing for linear, time-invariant, stable systems, but a few important points need to be highlighted. The Lyapunov inequalities (13) and (14) generalize the classical Lyapunov equations (2) and (3) and need to be solved uniformly in *x* in a given subset $\mathcal {S}$ of the state space. There are many ways to solve families of LMIs of the form (13) and (14). It is common practice to reduce families of LMIs like (13) and (14) to a finite family of LMIs through convex relaxation, see, e.g., (Boyd et al. 1994) and references therein. Another approach is to solve the inequalities (13) and (14) on a suitably dense grid covering the set $\mathcal {S}$, which then allows one to exploit the continuity properties of the Jacobian matrix $\partial f(x)$ to ensure that the inequalities are satisfied everywhere. This approach is analogous to a classical computational procedure for finding a controllability Gramian and an observability Gramian for a parameter-dependent system (see, e.g., Wood et al. 1996 and Son and Stykel 2017 for recent developments). An in-depth discussion on the computational aspects behind the solution families of LMIs of the form (13) and (14) is given in (Forni and Sepulchre 2019), Section VI). However, for the convenience of the reader, a heuristic procedure to select the parameters *p* and $\lambda (x)$ is given in Appendix A.

In contrast to classical balancing, (13) and (14) do not necessarily admit a solution. Moreover, if solutions do exist, the reachability and observability gramians are not necessarily positive definite, but have a fixed inertia. The existence of solutions for (13) and (14) ensures *p*-dominance of the system (8), since both (13) and (14), together with $BB^{\mathsf {T}} \ge 0$ and $C^{\mathsf {T}} C \ge 0$, directly imply (10).

In analogy with classical balancing, a change of coordinates ${\bar{x} = T^{-1} x}$ for system (8) naturally induces a change of coordinates ${\delta \bar{x} = T^{-1} \delta x}$ on its linearization (9). This, in turn, acts on the reachability gramian and the observability gramian as in (4). Differential balancing thus amounts to finding a transformation *T* which *simultaneously diagonalizes* the matrices *P* and *Q*. This problem admits a solution whenever the spectrum of *P* and *Q* satisfies certain assumptions (see, e.g., Uhlig 1973; Kenney and Hewer 1987; Therapos 1989). The solution of this problem and the corresponding algorithms are discussed in Appendix A.

Similar to classical balancing, the matrix $T\in \mathbb {R}^{n \times n}$ is said to be a *(principal-axis) balancing transformation* if (5) holds, in which case the corresponding representation in coordinates of system (8) is said to be *(principal-axis) balanced*. The diagonal elements of $\Sigma$ are said to be the *characteristic values* of the system.

#### Model reduction by differential balanced truncation

*Differential balanced truncation* for a *p*-dominant system (8) consists in eliminating, by truncation, the state variables corresponding to the least $n-r$ characteristic values (in absolute value), where ${r \le p}$ is the order of the reduced order model. To illustrate this method, assume that the Lyapunov inequalities (13) and (14) are solved by ${P\in \mathbb {R}^{n \times n}}$ and ${Q\in \mathbb {R}^{n \times n}}$, with ${\text {In}}(P) = {\text {In}}(Q) = (p,0,n-p)$. Further, assume that system (8) is (principal-axis) balanced, so that the gramians can be partitioned as

15 $$\begin{aligned} P = Q = \Sigma = \left[ \begin{array}{cc} \Sigma _1 &{} 0 \\ 0 &{} \Sigma _2 \end{array} \right] , \end{aligned}$$

with ${\Sigma _1 = {\text {diag}}(\sigma _1, \ldots , \sigma _r)}$ and ${\Sigma _2 = {\text {diag}}(\sigma _{r+1}, \ldots , \sigma _n)}$ such that ${{\text {In}}(\Sigma _1)=(p,0, r-p)}$ and ${{\text {In}}(\Sigma _2)=(0,0, n-r)}$, respectively. Then (15) directly induces the partitions $x = [\,x_1^{\mathsf {T}}\, x_2^{\mathsf {T}}\,]^{\mathsf {T}}$, with ${x_1 \in \mathbb {R}^r}$ and ${x_2 \in \mathbb {R}^{n-r}}$, and

16 $$\begin{aligned} f(x) = \left[ \begin{array}{c} f_1(x_1,x_2) \\ f_2(x_1,x_2) \end{array} \right] , ~ B = \left[ \begin{array}{c} B_1\\ B_2 \end{array} \right] , ~ C = \left[ \begin{array}{cc} C_1&C_2 \end{array} \right] . \end{aligned}$$

The reduced order model obtained by differential balanced truncation is defined by setting ${x_2 = 0}$ and discarding the dynamics of $x_2$, yielding

17 $$\begin{aligned} \quad \dot{\xi } = f_1(\xi ,0)+B_1 {\hat{u}}, \quad {\hat{y}}=C_1\xi , \end{aligned}$$

with $${\xi \in \mathbb {R}^r}$$, $${{\hat{u}}\in \mathbb {R}^m}$$, $${{\hat{y}}\in \mathbb {R}^l}$$.

The reduced order model (17) is (principal-axis) balanced and *p*-dominant with rate ${\hat{\lambda }}(\xi ) =\lambda (\xi ,0)$. To see this, consider the partition (16) and the Lyapunov inequalities (13) and (14) with ${x = [\,\xi ^{\mathsf {T}} \ 0^{\mathsf {T}}\,]^{\mathsf {T}}}$, which gives

18 $$\begin{aligned}&\partial f_1(\xi ,0) \Sigma _1\!+\!\Sigma _1\partial f_1(\xi ,0)^{\mathsf {T}}\!+\!2\lambda (\xi ,0) \Sigma _1 +B_1B_1^{\mathsf {T}}\!+\!\varepsilon I \!\le \! 0 , \end{aligned}$$

19 $$\begin{aligned}&\partial f_1(\xi ,0)^{\mathsf {T}}\Sigma _1\!+\!\Sigma _1\partial f_1(\xi ,0)\!+\!2\lambda (\xi ,0) \Sigma _1\!+\!C_1^{\mathsf {T}}C_1\!+\!\varepsilon I\!\le \!0 , \end{aligned}$$

and shows that (17) is (principal-axis) balanced. Moreover, both (18) and (19) imply

20 $$\begin{aligned} \partial f_1(\xi ,0)\Sigma _1+\Sigma _1\partial f_1(\xi ,0)^{\mathsf {T}}+2\lambda (\xi ,0)\Sigma _1+\varepsilon I\le 0 . \end{aligned}$$

By Definition 1, the reduced order model (17) is thus *p*-dominant with rate $\hat{\lambda }(\xi )= \lambda (\xi ,0)$, since ${\text {In}}(\Sigma _1)=(p,0, r-p)$.

## Results

We illustrate the proposed model reduction framework by approximating the behavior of a classical biological model of circadian oscillations: the Goldbeter model (Goldbeter 1995). Experiments have been conducted using standard routines of MATLAB (R2019b) on a 3.5 GHz Intel Core i7 processor.

### The Goldbeter model

The Goldbeter model is a classical model in cellular physiology which describes circadian oscillations in the expression of the gene *per* in *Drosophila* (Goldbeter 1995) (see also Keener and Sneyd 2008, p. 440). The model is governed by the equations

21a $$\begin{aligned} \dot{M}&= \tfrac{v_s K_I^n}{K_I^n+P_N^n} - \tfrac{v_mM}{k_m+M}, \end{aligned}$$

21b $$\begin{aligned} \dot{P}_0&= k_sM - \tfrac{V_1P_0}{K_1+P_0} + \tfrac{V_2P_1}{K_2+P_1}, \end{aligned}$$

21c $$\begin{aligned} \dot{P}_1&= \tfrac{V_1P_0}{K_1+P_0} -\tfrac{V_2P_1}{K_2+P_1} -\tfrac{V_3P_1}{K_3+P_1} + \tfrac{V_4P_2}{K_4+P_2}, \end{aligned}$$

21d $$\begin{aligned} \dot{P}_2&= \tfrac{V_3P_1}{K_3+P_1} - \tfrac{V_4P_2}{K_4+P_2} - k_1 P_2 + k_2 P_N - \tfrac{v_dP_2}{k_d+P_2}, \end{aligned}$$

21e $$\begin{aligned} \dot{P}_N&= k_1 P_2 -k_2 P_N , \end{aligned}$$

in which ${M\in \mathbb {R}_{+}}$ is the concentration of *per* mRNA, ${P_1\in \mathbb {R}_{+}}$, ${P_2\in \mathbb {R}_{+}}$ and ${P_3\in \mathbb {R}_{+}}$ are the concentrations of unphosphorylated, monophosphorylated and biphosphorylated PER protein, and ${P_N\in \mathbb {R}_{+}}$ is the concentration of PER protein in the nucleus, respectively. With the original parameters—reported in Table 1 for the reader’s convenience—the Goldbeter model has bounded solutions, a unique stable limit cycle and a unique unstable equilibrium point (Murray 2007).

**Table 1:** Parameter values of the Goldbeter model (Goldbeter 1995)

$v_s = 0.76\,(\mu \hbox {Mh}^{-1})$	$k_s = 0.38\,(\hbox {h}^{-1})$
$v_m = 0.65\,(\mu \hbox {Mh}^{-1})$	$k_1 = 1.9\,(\hbox {h}^{-1})$
$v_d = 0.95\,(\mu \hbox {Mh}^{-1})$	$k_2 = 1.3\,(\hbox {h}^{-1})$
$V_1 = 3.2\,(\mu \hbox {Mh}^{-1})$	$K_{d} = 0.2\,(\mu \hbox {M})$
$V_2 = 1.58\,(\mu \hbox {Mh}^{-1})$	$K_{I} = 1\,(\mu \hbox {M})$
$V_3 = 5\,(\mu \hbox {Mh}^{-1})$	$K_{m} = 0.5\,(\mu \hbox {M})$
$V_4 = 2.5\,(\mu \hbox {Mh}^{-1})$	$K_{1,2,3,4} = 2\,(\mu \hbox {M})$

### Dominance analysis

To illustrate our model reduction framework, we consider system (21) with an added exogenous input *u* to (21a) and with $P_N$ as the output variable *y*, namely

22a $$\begin{aligned} \dot{M}&= \tfrac{v_s K_I^n}{K_I^n+P_N^n} - \tfrac{v_mM}{k_m+M} + u, \end{aligned}$$

22b $$\begin{aligned} \dot{P}_0&= k_sM - \tfrac{V_1P_0}{K_1+P_0} + \tfrac{V_2P_1}{K_2+P_1}, \end{aligned}$$

22c $$\begin{aligned} \dot{P}_1&= \tfrac{V_1P_0}{K_1+P_0} -\tfrac{V_2P_1}{K_2+P_1} -\tfrac{V_3P_1}{K_3+P_1} + \tfrac{V_4P_2}{K_4+P_2}, \end{aligned}$$

22d $$\begin{aligned} \dot{P}_2&= \tfrac{V_3P_1}{K_3+P_1} - \tfrac{V_4P_2}{K_4+P_2} - k_1 P_2 + k_2 P_N - \tfrac{v_dP_2}{k_d+P_2}, \end{aligned}$$

22e $$\begin{aligned} \dot{P}_N&= k_1 P_2 -k_2 P_N , \end{aligned}$$

22f $$\begin{aligned} y&= P_N , \end{aligned}$$

which can be described as a system of the form (8) by defining ${x = [\, M \, P_0 \, P_1 \, P_2 \, P_N \,]^{\mathsf {T}}} \in \mathbb {R}^{5}$ and

23a $$\begin{aligned} f(x)&\!= \! \! \left[ \begin{array}{c} \frac{v_s K_I^n}{K_I^n+x_5^n} - \frac{v_m x_1}{k_m+x_1} \\ k_s x_1 - \frac{V_1 x_2}{K_1+x_2} + \frac{V_2 x_3}{K_2+x_3} \\ \frac{V_1x_2}{K_1+x_2} - \frac{V_2x_3}{K_2+x_3} - \frac{V_3x_3}{K_3+x_3} + \frac{V_4x_4}{K_4+x_4} \\ \frac{V_3x_3}{K_3+x_3} - \frac{V_4 x_4}{K_4+x_4} - k_1 x_4 + k_2 x_5 - \frac{v_d x_4}{k_d+x_4} \\ k_1 x_4 -k_2 x_5 \\ \end{array} \right] \! \! ,\end{aligned}$$

23b $$\begin{aligned} B&= \left[ \begin{array}{ccccc} 1&0&0&0&0\end{array} \right] ^{\mathsf {T}}, \end{aligned}$$

23c $$\begin{aligned} C&= \left[ \begin{array}{ccccc} 0&0&0&0&1 \end{array} \right] . \end{aligned}$$

24 $$\begin{aligned} \partial f(x) = \left[ \begin{array}{ccccc} -\frac{v_m k_m}{(k_m + x_1)^2} &{} 0 &{} 0 &{} 0 &{} - \frac{ n v_s K_I^n x_5^{n-1}}{(K_I^n + x_5^n)^2} \\ k_s &{} -\frac{V_i1K_1 }{(K_1 + x_2)^2} &{} \frac{V_2 K_2 }{(K_2 + x_3)^2} &{} 0 &{} 0 \\ 0 &{} \frac{V_1K_1 }{(K_1 + x_2)^2} &{} -\frac{V_2 K_2 }{(K_2 + x_3)^2}-\frac{V_3 K_3 }{(K_3 + x_3)^2} &{} \frac{V_4 K_4 }{(K_4 + x_4)^2} &{} 0 \\ 0 &{} 0 &{} \frac{V_3 K_3 }{(K_3 + x_3)^2} &{} -k_1 -\frac{V_4 K_4 }{(K_4 + x_4)^2} -\frac{v_d k_d}{(k_d + x_4)^2} &{} k_2 \\ 0 &{} 0 &{} 0 &{} k_1 &{} -k_2 \\ \end{array} \right] , \end{aligned}$$

System (22) has been simulated over the time interval $[t_i, t_f]$, with ${t_i = 0 \ \text {[ \!h\! ]}}$ and ${t_f = 100 \ \text {[ \!h\! ]}}$, selecting a zero initial condition and ${u=0}$. The solution has been sampled over a fine grid of points in a neighborhood of the limit cycle $\mathcal {S}$, taking ${N = 75}$ distinct samples $x(t_k)$ of the solution at steady state at equally spaced time instants $t_k \in [t_1, t_{N}]$, with ${t_1 = 87 \ \text {[ \!h\! ]}}$ and ${t_{N} = 100 \ \text {[ \!h\! ]}}$.

**Fig. 1:**
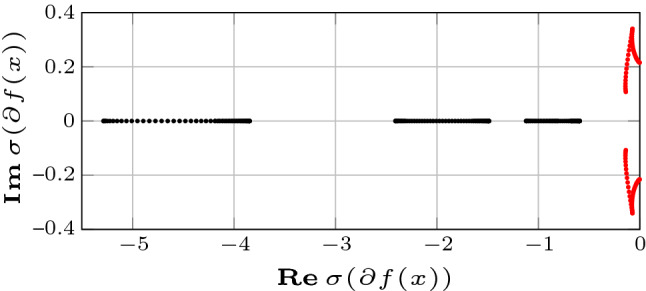
Eigenvalues of the Jacobian of the system (22) at distinct samples $\{x(t_k)\}_{k=1}^N$ of the solution at steady state. Note that $\partial f(x)$ has 2 eigenvalues with real part larger than ${-\lambda (x)}$ (red) and 3 eigenvalues with real part less than ${-\lambda (x)}$ (black) for ${\lambda (x) = k_1 x_4-k_2x_5-0.4}$

The dominance properties of system (22) have been analyzed selecting the parameters *p* and $\lambda (x)$ by means of the heuristic procedure given in Appendix A, which, taking the limit cycle $\mathcal {S}$ as the region of interest, yields ${p=2}$ and ${\lambda (x) = k_1 x_4-k_2x_5-0.4.}$ Figure 1 shows the eigenvalues of the Jacobian of system (22)—given in (24)—at distinct samples $\{x(t_k)\}_{k=1}^N$ of the solution at steady state. Note that $\partial f(x)$ has 2 eigenvalues with real part larger than ${-\lambda (x)}$ (red) and 3 eigenvalues with real part less than ${-\lambda (x)}$ (black) for $\lambda (x) = k_1 x_4-k_2x_5-0.4$, which suggests the existence of a dominant splitting.

By evaluating the Jacobian of the system $\partial f(x)$ at distinct samples $\{x(t_k)\}_{k=1}^N$ of the solution at steady state, the LMI (10) has been solved for ${\varepsilon = 0.001}$ and $\lambda (x) = k_1 x_4-k_2x_5-0.4$, yielding

25 $$\begin{aligned} P = \left[ \begin{array}{rrrrr} -1.028 &{} -1.795 &{} 0.894 &{} 0.730 &{} 0.128 \\ -1.795 &{} 2.269 &{} -3.279 &{} -1.944 &{} 0.535 \\ 0.894 &{} -3.279 &{} 7.863 &{} -4.262 &{} -0.663 \\ 0.730 &{} -1.944 &{} -4.262 &{} 10.561 &{} -4.572 \\ 0.128 &{} 0.535 &{} -0.663 &{} -4.572 &{} 1.992 \end{array} \right] , \end{aligned}$$

whose the inertia is ${\text {In}}(P) = (2,0,3)$. The same analysis has been repeated for different sampling times obtaining similar results. In agreement with Theorem 1, this suggests that the Goldbeter model (22) is 2-dominant with rate ${\lambda (x) = k_1 x_4-k_2x_5-0.4}$ in a neighborhood of the limit cycle $\mathcal {S}$.

### Differential balanced truncation

We now turn to the question of building a reduced order model of system (22). First, the reachability and observability gramians have been computed by sampling the Jacobian of the system as above and solving for ${\varepsilon = 0.001}$ and ${\lambda (x) = k_1 x_4-k_2x_5-0.4}$ the LMIs (13) and (14), which yields the solutions

26 $$\begin{aligned} P \! = \! \left[ \begin{array}{rrrrr} -30.284 &{}-42.356 &{} -12.762 &{} 8.616 &{} 27.678 \\ -42.356 &{} 10.001 &{} -7.797 &{} -22.240 &{} -38.751 \\ -12.762 &{}-7.797 &{} 8.157 &{} -9.426 &{} -24.427 \\ 8.616 &{}-22.240 &{} -9.426 &{} 1.1810 &{} -6.579 \\ 27.678 &{} -38.751 &{} -24.427 &{} -6.579 &{} 10.946 \end{array} \right] \!\nonumber \\ \end{aligned}$$

and

27 $$\begin{aligned} Q \! = \! \left[ \begin{array}{rrrrr} -7.463 &{} -18.749 &{} -2.445 &{} 7.607 &{} 13.193 \\ -18.749 &{} 10.261 &{} -11.346 &{} -15.919 &{} -16.251 \\ -2.445 &{}-11.346 &{} -2.357 &{} -6.946 &{} -9.414 \\ 7.607 &{} -15.919 &{}-6.946 &{} -0.179 &{} -3.875 \\ 13.193 &{} -16.251 &{} -9.414 &{} -3.875 &{} -0.263 \end{array} \right] , \! \end{aligned}$$

whose inertia is ${\text {In}}(P) = {\text {In}}(Q) = (2,0,3)$.

The spectrum of the matrix *PQ* is real and positive: ${\sigma ({PQ})} = \{\, 2847.3, \, 1590.1, \, 984.4, \, 161.3, \, 21.6 \, \}$. The system (22) thus admits a (principal-axis) balancing transformation by virtue of Theorem 2 (given in Appendix A). A (principal-axis) balancing transformation ${T\in \mathbb {R}^{5\times 5}}$ has been computed using Algorithm 1 (also given in Appendix A), which yields

28 $$\begin{aligned} T = \left[ \begin{array}{rrrrr} 0.562 &{} 0.924 &{} 0.042 &{} 0.160 &{} -0.406 \\ 0.948 &{} 0.090 &{} -0.097 &{} 0.493 &{} 0.897 \\ 0.399 &{} -0.128 &{} 0.459 &{} -0.899 &{} 0.267 \\ 0.280 &{} -0.347 &{} -0.968 &{} -0.179 &{} -0.249 \\ 0.351 &{} -0.682 &{} 0.536 &{} 0.690 &{} -0.658 \end{array} \right] . \end{aligned}$$

The change of coordinates ${\bar{x} = T^{-1} x}$ simultaneously diagonalizes the gramians according to (4), yielding

29 $$\begin{aligned} \bar{P} \! = \! \bar{Q} \! = \! \! \left[ \begin{array}{ccccc} -39.877 &{}0 &{}0 &{}0 &{}0 \\ 0 &{}-31.376 &{}0 &{}0 &{}0 \\ 0 &{}0 &{}4.651 &{}0 &{}0 \\ 0 &{}0 &{}0 &{} 12.699 &{}0 \\ 0 &{}0 &{}0 &{}0 &{}53.360 \end{array} \right] \! \! . \end{aligned}$$

**Fig. 2:**
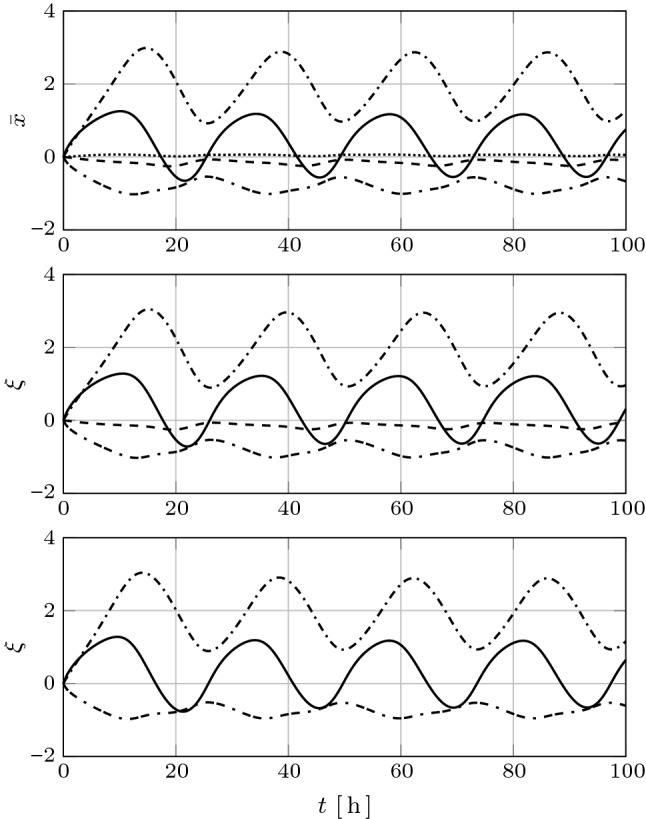
Top: Time history of the solutions of system (22), with ${u=0}$, in the new coordinates (top) and those of the reduced order models of order ${r=4}$ (middle) and ${r=3}$ (bottom) obtained using differential balanced truncation, respectively

For ${\varepsilon = 0.001}$ and ${\lambda (x) = k_1 x_4-k_2x_5-0.4}$, the characteristic values of the system are thus $\sigma _1 = -39.877$, $\sigma _2 = -31.376$, $\sigma _3 = 4.651$, $\sigma _4 = 12.699$, $\sigma _5 = 53.360$. Note that ${\sigma _3}$ and ${\sigma _4}$ are much smaller in absolute value relative to ${\sigma _1}$, ${\sigma _2}$ and ${\sigma _5}$, which suggests that the corresponding state variables ${\bar{x}_3}$ and ${\bar{x}_4}$ have a negligible effect on the overall (differential) input–output behavior and, hence, may be eliminated. This intuition is confirmed by Fig. 2, which shows the time history of the solutions of system (22), with ${u=0}$, in the new coordinates $\bar{x}$ (top) as well as those of the reduced order models of order ${r=4}$ (middle) and ${r=3}$ (bottom) obtained using differential balanced truncation, respectively. Note that differential balanced truncation indeed eliminates the state variables which, in the new coordinates, have the least variation relative to the other state variables.

**Fig. 3:**
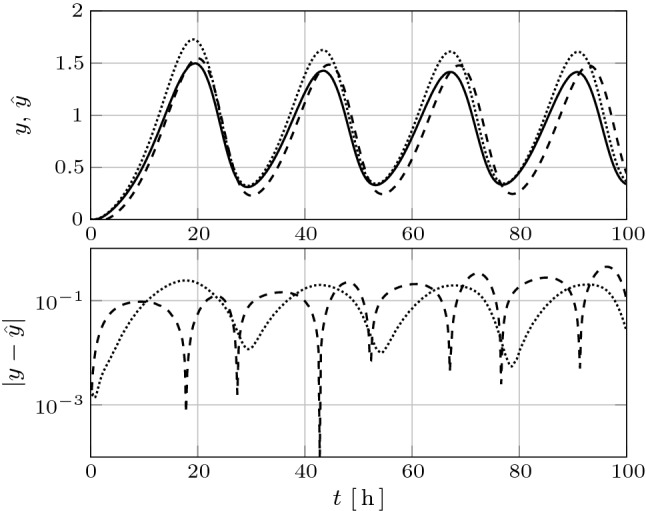
Top: Time history of the output of system (22), with ${u=0}$, (solid) and those of the reduced order models of order ${r=4}$ (dotted) and ${r=3}$ (dashed) obtained using differential balanced truncation, respectively. Bottom: Time history of the corresponding output errors in absolute value (logarithmic scale)

Figure 3 (top) shows the time history of the output of system (22), with ${u=0}$, in the new coordinates (solid) and of the reduced order models of order ${r=3}$ (dotted) and ${r=4}$ (dashed) obtained using differential balanced truncation, as well as those of the corresponding errors in absolute value (bottom), respectively. Note that while the output of the reduced order model of order ${r=3}$ is out of phase with the oscillation of the original system, the output of the reduced order model of order ${r=4}$ tracks well the output of the original system.

**Fig. 4:**
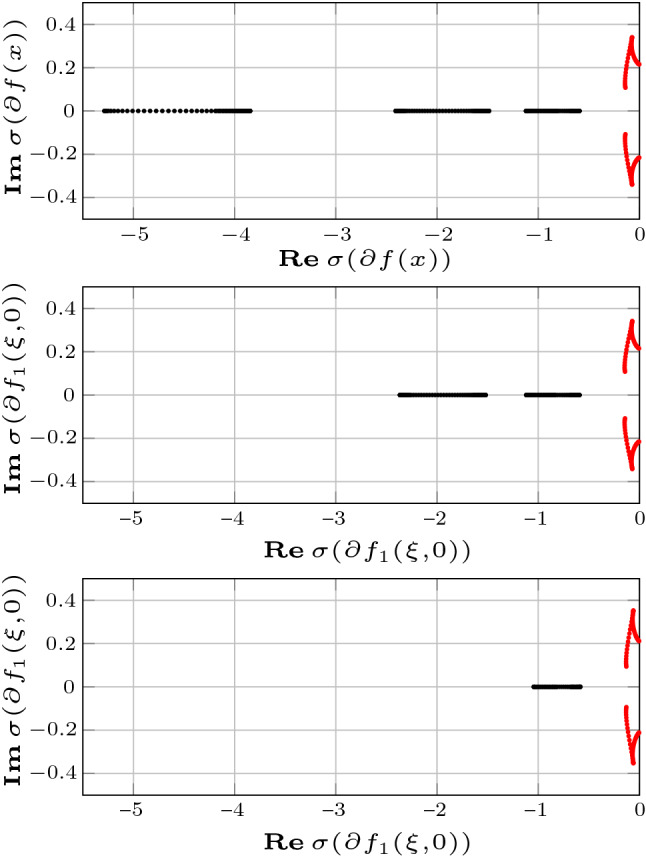
Fig. 4 shows the eigenvalues of the Jacobian of the system (22), with ${u=0}$, at distinct samples $x(t_k)$ of the solution at steady state (top) and those of the corresponding reduced order models of order ${r=4}$ (middle) and ${r=3}$ (bottom) obtained by differential balanced truncation. The figure suggests that each reduced order model preserves 2-dominance with rate $\lambda (x)$. It is interesting to note that differential balanced truncation eliminates the modes associated with the most negative eigenvalues of the linearized dynamics. This is consistent with the intuition that a “good” reduced order model should capture the dominant dynamics and, hence, eliminate the fastest transient modes of the (differential) input–output behavior of the system. Eigenvalues of the Jacobian of the system (22), with $u=0$, at distinct samples $x(t_k)$ of the solution at steady state (top) and those of the corresponding reduced order models of order ${r=4}$ (middle) and ${r=3}$ (bottom) obtained by differential balanced truncation, respectively

## Discussion

The proposed model reduction framework offers a series of benefits. First, the construction of reduced order models relies on standard convex optimization and linear algebra tools, namely the solution of LMIs and the simultaneous diagonalization of a pair of matrices. This renders model reduction easy to implement and favors tractability. Second, the quality of a reduced order model can be interpreted in *quantitative* terms using classical control-theoretic notions and tools, such as eigenvalues, Nyquist diagrams and Bode diagrams. Third, our approach is compositional and compatible with open systems modeling. Combining our model reduction framework with the small-gain theorem for *p*-dominance (Forni and Sepulchre 2019), the global emergent behavior of a complex biological system can be approximated using reduced order models of its elementary components.

On the other hand, the proposed model reduction framework can be improved in several ways. A first limitation of our approach is that it relies on a change of coordinates which, in general, does not preserve the biological meaning of state space variables. This is a well-known drawback of model reduction methods based on balanced truncation (Snowden et al. 2017), which can be circumvented by imposing a given sparsity pattern while computing the reachability and observability gramians as discussed, e.g., in Sootla and Anderson (2017). A similar approach can be taken within our framework to maintain the biological interpretation of state space variables by requiring that the Lyapunov inequalities (13) and (14) have a given sparsity pattern.

A second limitation is that the LMIs (13) and (14) are only verified in a neighborhood of the limit cycle, resulting in a local result. While our analysis is not infinitesimal and can be in principle adapted to any desired region of the state space, it would be of interest to compare the proposed results to the model reduction of a periodic linear system obtained by linearizing the nonlinear dynamics along the limit cycle. Model reduction for periodic linear systems has been addressed, e.g., in Varga (2000); Sandberg and Rantzer (2004).

A third limitation of our approach is that it only applies to systems of moderate size. Indeed, the dimension of a system directly influences the number of variables and LMIs required to solve (13) and (14), which is limited to a few thousands in currently available solvers. Nonetheless, the compositional nature of our framework enables one to partition a large-scale system into smaller subsystems, compute reduced order models for each individual subsystem and finally obtain by interconnection an overall reduced order model, the behavior of which can be characterized *a posteriori* using the small gain for *p*-dominance (Forni and Sepulchre 2019).

A further limitation concerns the computational cost of the simulation of the reduced order model (17). Given a balancing transformation ${T\in \mathbb {R}^{n \times n}}$, the simulation of the reduced order model (17) requires the evaluation of $f_1(\xi ,0) = \bar{S}_1f({S}_1 \xi )$, where ${S}_1$ are the first *r* columns of *T* and $\bar{S}_1$ are the first *r* rows of $T^{-1}$, respectively. This means that, in general, the computational cost of evaluating $f_1(\xi ,0)$ depends on the order *n* of the original system. As a result, the reduced order model may offer a limited performance speed-up compared to the original model. This computational issue has been previously addressed in the literature (see, e.g., Chaturantabut and Sorensen 2010). Nonlinear functions can be projected onto a subspace that approximates the space generated by the nonlinear terms and that is spanned by a basis of lower dimension (see Chaturantabut and Sorensen 2010, Section 2.2 for further detail). The same strategy can be used to reduce the computational cost of the simulation of the reduced order model (17), since our framework also relies on constant projections.

**Fig. 5:**
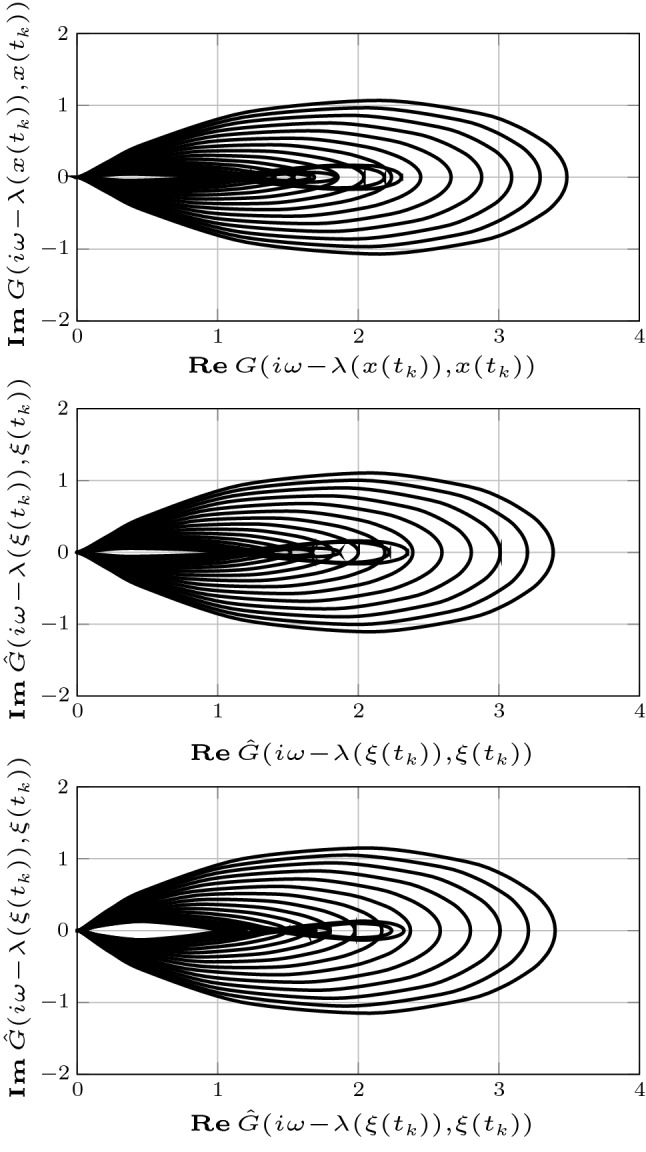
Nyquist diagram of the family of transfer functions (30) and (31) associated with the linearizations of the original system (top) and the reduced order models of order ${r=4}$ (middle) and ${r=3}$ (bottom)

**Fig. 6:**
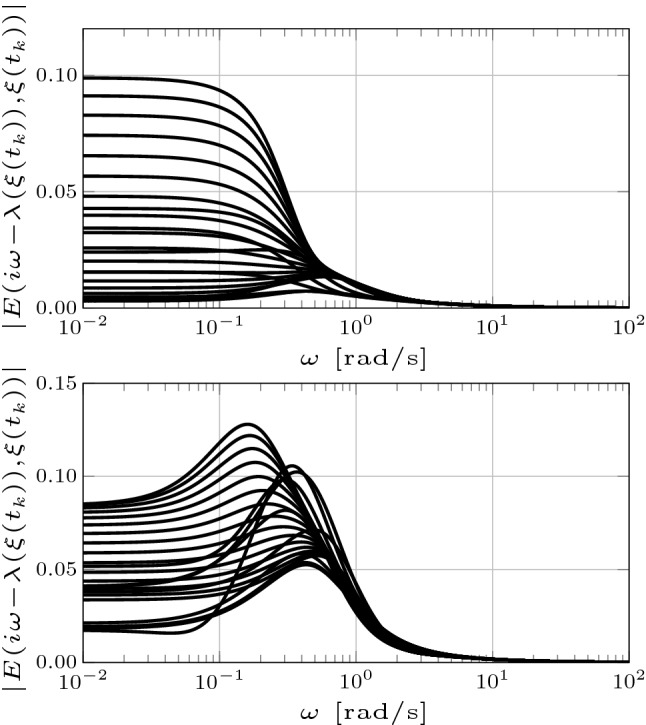
Bode diagram of the family of error transfer functions (32) associated with the reduced order models of order ${r=4}$ (top) and ${r=3}$ (bottom)

Finally, our framework does not provide an *a priori* error bound. Similar to the case of linear time-invariant stable systems, it is reasonable to expect that the approximation error is bounded by a function of the neglected characteristic values. A thorough analysis of this issue is beyond the scope of this preliminary study and is the subject of ongoing research. Nonetheless, we observe that in the context of our example the differential input–output behavior of the original system is well approximated. This can be appreciated by considering the Nyquist diagrams of the family of transfer functions defined by the linearization of the original system

30 $$\begin{aligned} G(s,x(t_k)) = C(s- \partial f(x(t_k)))^{-1} B , \end{aligned}$$

as well as those of the family of transfer functions defined by the linearization of each reduced order model

31 $$\begin{aligned} {\hat{G}}(s,\xi (t_k)) = {\hat{C}}(s- \partial f_1(\xi (t_k),0))^{-1} {\hat{B}} , \end{aligned}$$

which are formed by tracing ${s \in \mathbb {C}}$ around the Nyquist “D contour” consisting of the imaginary axis combined with an arc at infinity connecting the endpoints of the imaginary axis (see Åström and Murray 2020 for further detail). Nyquist diagrams are widely employed in control theory (Åström and Murray 2020) and provide considerable insight into the input–output behavior of a dominant system (Miranda-Villatoro et al. 2018; Padoan et al. 2019a, b). Figure 5 shows the Nyquist diagrams of the family of transfer functions (30) and (31) defined by the original system (top) and by the reduced order models of order ${r=4}$ (middle) and ${r=3}$ (bottom), respectively. Not only the Nyquist diagrams of each member of the family of transfer functions (30) and (31) have a similar shape, but the *distance* between one another is negligible. The quality of the approximation is reflected in Fig. 6, which shows the Bode diagrams the magnitude of the family of error transfer functions defined as

32 $$\begin{aligned} E(s,\xi (t_k)) = G(s,x(t_k)) - {\hat{G}}(s,\xi (t_k)) . \end{aligned}$$

Note that the approximation the error is bounded as

33 $$\begin{aligned} |E(s,\xi (t_k))| < 0.15 ,\quad \end{aligned}$$

for the reduced order model of order $r=4$ and as

34 $$\begin{aligned} |E(s,\xi (t_k))| < 0.2 ,\quad \end{aligned}$$

for the reduced order model of order $r=3$, which indicates that the differential input–output behavior of the original system is well approximated by that of both reduced order models.

## Conclusion

The paper has outlined a model reduction framework for biological systems whose behavior is not restricted to the stability of a single equilibrium. Classical balanced truncation for linear stable systems has been extended to dominant nonlinear systems using differential analysis. The asymptotic behavior of reduced order models has been characterized by ensuring that the property of *p*-dominance is preserved. This approach is tractable and offers quantitative tools to predict the behavior of a reduced order model *a priori*. Preliminary numerical results suggest that the proposed model reduction framework may be relevant to the approximation of a wide range of biological systems.

## A Algorithms

This section provides a heuristic procedure for the selection of the parameters *p* and $\lambda (x)$ in dominance analysis as well as necessary and sufficient conditions for the existence of a (principal-axis) balancing transformation and an algorithm to find such a transformation.

### A.1 Selecting *p* and $\lambda (x)$ for dominance analysis

A necessary condition for *p*-dominance of the system (8) is that the spectrum of the family of matrices $\partial f(x) + \lambda (x)I$ admits a uniform splitting into *p* eigenvalues with positive real part and $n-p$ eigenvalues with negative real part (Forni and Sepulchre 2019, Section VI). The analysis of the spectrum of the Jacobian matrix $\partial f(x)$ can therefore inform the selection of the parameters *p* and $\lambda (x)$. In particular, the following heuristic procedure can been used to estimate *p* and $\lambda (x)$ based on the evaluation of the Jacobian matrix $\partial f(x)$ over a sufficiently fine grid of points $\{x_k\}_{k=1}^N$, with ${x_k \in \mathcal {S}}$ and a subset $\mathcal {S} \subset \mathbb {R}^n$ of interest.

1. For each ${k = 1, \ldots , N}$, compute the eigenvalues $\lambda _{k,1}, \ldots , \lambda _{k,n}$ of the matrix $\partial f(x_k)$.
2. Select an integer *p* such that
  $$\begin{aligned}{\mathbb {Re} \lambda _{k,1} \ge \cdots \ge \mathbb {Re} \lambda _{k,p} > \mathbb {Re} \lambda _{k,p+1} \ge \cdots \ge \mathbb {Re} \lambda _{k,n}}.\end{aligned}$$
3. For each ${k = 1, \ldots , N}$, define
  $$\begin{aligned}{\bar{\lambda }}_k = -\min \left( \tfrac{\mathbb {Re} \lambda _{k,p}+\lambda _{k,p+1}}{2},0\right) .\end{aligned}$$
4. Estimate $\lambda (x)$ by fitting $\{\bar{\lambda }_k\}_{k=1}^{N}$ to the samples $\{x_k\}_{k=1}^{N}$.

The heuristic procedure outlined above comprises four basic steps. The first step consists in computing the spectrum of the Jacobian matrix $\partial f(x_k)$ at each point $x_k$. The second step consists in estimating the parameter *p* based on the existence of a splitting into *p* “dominant” eigenvalues ${\lambda _{k,1}, \ldots , \lambda _{k,p}}$ and $n-p$ “transient” eigenvalues ${\lambda _{k,p+1}, \ldots , \lambda _{k,n}}$. Note that if such splitting does not exist, the system is not *p*-dominant for any *p* by the necessary condition mentioned above. The third step defines ${\bar{\lambda }}_k$ as the negative part of the midpoint between $\mathbb {Re} \lambda _{k,p}$ (the real part of the smallest dominant eigenvalue) and $\mathbb {Re} \,\lambda _{k,p+1}$ (the real part of the largest transient eigenvalue). Finally, an estimate of the function $\lambda (x)$ is computed by fitting $\{\bar{\lambda }_k\}_{k=1}^{N}$ to the samples $\{x_k\}_{k=1}^{N}$.

### A.2 Balancing gramians with inertia

Recall that the goal of differential balancing for system (8) boils down to finding a transformation ${T\in \mathbb {R}^{n \times n}}$ which simultaneously diagonalizes the reachability gramian $P\in \mathbb {R}^{n \times n}$ and the observability gramian $Q\in \mathbb {R}^{n \times n}$, defined implicitly by the Lyapunov inequalities (13) and (14). The following result provides necessary and sufficient conditions for the existence of a (principal-axis) balancing transformation *T*. The proofs are omitted as the claim follows directly from (Uhlig 1973), Corollary 1.4 see also (Kenney and Hewer (1987), Theorems 1 and 2) and (Therapos 1989).

#### Theorem 2

Suppose (13) and (14) admit solutions $P\in \mathbb {R}^{n \times n}$ and $Q\in \mathbb {R}^{n \times n}$, with ${{\text {In}}(P)= {\text {In}}(Q) = (p,0,n-p)}$. Then there exists a (principal-axis) balancing transformation if and only if the product *PQ* is similar to a (positive) real diagonal matrix.

#### Remark 1

We emphasize that not all *p*-dominant systems can be (principal-axis) balanced for a given rate $\lambda$, as illustrated by the following example from (Kenney and Hewer 1987). Consider system (1), with

35 $$\begin{aligned} A = \left[ \begin{array}{rr} -1 &{} 1 \\ 0 &{} 2 \end{array} \right] , \ B = \left[ \begin{array}{rr} 1 &{} 0 \\ 0 &{} 1 \end{array} \right] , \ C = \left[ \begin{array}{rr} 1 &{} 0 \\ 0 &{} 1 \end{array} \right] . \end{aligned}$$

The reachability gramian and the observability gramian of the system with rate $\lambda \equiv 0$ are

36 $$\begin{aligned} P = \left[ \begin{array}{rr} 3/4 &{} 1/4\\ 1/4 &{} - 1/4 \end{array} \right] , \quad Q = \left[ \begin{array}{rr} 1/2 &{} -1/2\\ -1/2 &{} 0 \end{array} \right] . \end{aligned}$$

The system is thus 1-dominant with rate ${\lambda \equiv 0}$, since ${\text {In}}(P) = {\text {In}}(Q) = (1,0,1)$. However, the gramians *P* and *Q* *cannot* be balanced, since ${\sigma ({PQ})} = \{ 1/16 \pm i \sqrt{15/16}\}$ and, hence, the condition given in Theorem 2 is violated. Nonetheless, the reachability gramian and the observability gramian of the system with rate ${\lambda \equiv 1.8}$ are

37 $$\begin{aligned} P = \left[ \begin{array}{rr} -0.0720 &{} -0.6944\\ -0.6944 &{} -1.6667 \end{array} \right] , \quad Q = \left[ \begin{array}{rr} 0.1852 &{} 0.0772\\ 0.0772 &{} -1.9239 \end{array} \right] , \end{aligned}$$

which shows that the system is 1-dominant with rate $\lambda \equiv 1.8$, since ${\text {In}}(P) = {\text {In}}(Q) = (1,0,1)$. In agreement with Theorem 2, the gramians *P* and *Q* *can* be (principal-axis) balanced, since ${\sigma ({PQ})} = \{ 0.0431, 3.0428 \}$. $\bigtriangleup$

We now provide a procedure to compute a (principal-axis) balancing transformation $T\in \mathbb {R}^{n \times n}$, described in pseudo-code in Algorithm 1. The procedure comprises four basic steps. The first step (line 1) computes the eigenvalue decomposition $S^{-1} PQS = \Sigma ^2$ of the product *PQ*. The second step (line 2) defines the matrices $X = S^{\mathsf {T}} Q S$ and $Y = S^{-1} P S^{-\mathsf {T}}$, which can be simultaneously diagonalized since $XY = YX$. The third step (line 3) computes a (unitary) coordinate transformation *U* which simultaneously diagonalizes *X* and *Y*, yielding $X = U\Lambda _o U^{\mathsf {T}}$ and $Y = U\Lambda _r U^{\mathsf {T}}$. Finally, a (principal-axis) balancing transformation is computed as $T = S U (\Lambda _r \Lambda _o^{-1})^{1/4}$ (line 4).

The following result can be proved by direct computation.

#### Theorem 3

Suppose (13) and (14) admit solutions $P\in \mathbb {R}^{n \times n}$ and $Q\in \mathbb {R}^{n \times n}$, with ${{\text {In}}(P)= {\text {In}}(Q) = (p,0,n-p)}$, such that the product *PQ* is similar to a (positive) real diagonal matrix. Then the output of Algorithm 1 is a (principal-axis) balancing transformation.

## Footnotes

1 $\mathcal {L}_2$ is the space of all measurable functions ${v:\mathbb {R}_{+}\rightarrow \mathbb {R}^q}$ with finite $\mathcal {L}_2$ norm, defined as ${\left\Vert v\right\Vert _{2} = \left( \int _{0}^{\infty } | v(\tau ) |^{2} d\tau \right) ^{1/2}}.$

2 To streamline the exposition, we often tacitly abuse terminology and omit “strict” throughout the paper.
